# Bibliography

**Published:** 1901-06

**Authors:** 


### Bibliography.

Our Teeth and how to take care of Them. By Victor C. Bell, M.D., D.D.S. Young America Publishing Company, 111 Fifth Avenue, New York.

This book has been prepared by Dr. Bell for the use of young children as a text-book in our schools. For this purpose it seems well adapted, and care has been taken to make it interesting through carefully prepared illustrations. The book is well arranged in short chapters, followed by a series of questions.

For the age, presumably, from six to ten years this little primer would be all that would be necessary, but for children of larger growth and more cultivated understanding something more should be given.

It is a gratification, however, to find that the trend of educational thought is towards the training of the young mind to the care of the body. The great effort heretofore has been the cultivation of the mental powers, permitting the health to be looked after by the medical attendant.

The time must come when the medical man and the dentist will be required as an essential part of the staff of all well-regulated schools. Sanitary science should be a necessary part of the curriculum, and in this should be included the care of the oral cavity.

This little book is, therefore, welcomed as an entering wedge to force the conservative element asunder and admit into our public-school system dental teaching, to include all that is important for a young person to know regarding the mouth as a whole.

If the world is ever to be educated to the importance of the teeth and the intimate relation the mouth holds to the entire system, it must be through the education of the young, not exclusively under the parental roof, but in the schools of the country. The quicker this great fact is learned by our school-boards the better it will be for the health and intelligence of future generations.

Poems of the Farm, and other Poems. By Charles Nelson Johnson. Chicago: Daniels Company Press, 1901.

Poetry and dental science have not been in harmony for at least two generations. In the earlier dental periodicals the searcher into dental history will find occasionally the older dentists dropping the ordinary prose for what they pleased to denominate poetry, and even to-day some of our trade contemporaries try to give a flavor of sentimentalism to the commercial side of these journals by a touch of doggerel verse.

It has, however, been left for a prominent dentist of the year 1901 to give us a genuine book of poems which appeal to two phases of human nature, the humorous and the better life of our common humanity.

The volume is modestly presented by the author without, as he says, "the slightest pretension to merit. In truth, they are not, strictly speaking, presented to the public at all." With the first statement very few readers will agree. To the writer they not only possess merit, but it is a question whether they do not equal some of the best in our literature.

The dialect poems relating to farm life fill up nearly one-half of the volume. The temptation to quote portions of several can scarcely be resisted, but space will not permit. To one sated with the menus of the club the poem dedicated to "An Old Boiled Dinner" will specially appeal. The author says,—

> "You may talk about your ices
> Filled with gastronomic vices,
> You may talk about your soups so thin—or thinner,
> On your menu's polyglot;
> But there's nothing hits the spot
> Like a steaming, fragrant, old boiled dinner."

That the author lived on a farm in his boyhood days is very evident, for no one could write so feelingly of farm work and "Doin' Chores on the Farm," unless he had worked from "Diggin' Turnips in the Fall" until the last spear of oats had been ingathered, or until, as the author expresses it, "I have raked and bound in harvest till I wisht 'at I was dead." Many, doubtless, can find this description one of the treasures of memory:

> "In the mornin' bright and early, just before the break o' day,
> Crawlin' out from under blankets where you'd give the world to stay,
> Tuggin' at a pair o' cowhides frozen stiff from overnight,
> Pullin', strainin', stampin', shiverin' in the dim, uncertain light."

One of the most characteristic pieces in the collection, and true to the life it is intended to represent, is the one entitled "The

Horse-Race at the Corners." It is worthy of reproduction here, for, while the critic might not call it poetry, it belongs to that class of rhymes that made "The One-Horse Shay" and Oliver Wendell Holmes equally famous.

The author's love of children is presented in the closing pages of this interesting volume. The poems here are the genuine outpouring of a heart full of love for the little ones expressed in this verse:

> "What is home without the children?
> Just an egg without the meat—
> Just a nut without the kernel—
> Just a sun without the heat."

That the author has found the time and inclination, after the absorbing and exhausting work of his profession, to cultivate the higher inspirations is an evidence that the spirit of man will at times overcome the shackles of environment and rise superior to the daily unpoetical duties of professional life.

